# Spatial clusters distribution and modelling of health care autonomy among reproductive‐age women in Ethiopia: spatial and mixed‐effect logistic regression analysis

**DOI:** 10.1186/s12913-020-06052-1

**Published:** 2021-01-20

**Authors:** Getayeneh Antehunegn Tesema, Yigizie Yeshaw, Ayenew Kasie, Alemneh Mekuriaw Liyew, Achamyeleh Birhanu Teshale, Adugnaw Zeleke Alem

**Affiliations:** 1 Department of Epidemiology and Biostatistics, Institute of Public Health, College of Medicine and Health Sciences, University of Gondar, Gondar, Ethiopia; 2 Department of Physiology, School of Medicine, College of Medicine and Health Sciences, University of Gondar, Gondar, Ethiopia; 3 Department of Health Education and Behavioral Science, Institute of Public Health, College of Medicine and Health Sciences and comprehensive specialized hospital, University of Gondar, Gondar, Ethiopia

**Keywords:** Mixed effect analysis, Spatial analysis, Geographic Information System

## Abstract

**Background:**

While millions of women in many African countries have little autonomy in health care decision-making, in most low and middle-income countries, including Ethiopia, it has been poorly studied. Hence, it is important to have evidence on the factors associated with women’s health care decision making autonomy and the spatial distribution across the country. Therefore, this study aimed to investigate the spatial clusters distribution and modelling of health care autonomy among reproductive-age women in Ethiopia.

**Methods:**

We used the 2016 Ethiopian Demographic and Health Survey (EDHS) data for this study. The data were weighted for design and representativeness using strata, weighting variable, and primary sampling unit to get a reliable estimate. A total weighted sample of 10,223 married reproductive-age women were included in this study. For the spatial analysis, Arc-GIS version 10.6 was used to explore the spatial distribution of women health care decision making and spatial scan statistical analysis to identify hotspot areas. Considering the hierarchical nature of EDHS data, a generalized linear mixed-effect model (mixed-effect logistic regression) was fitted to identify significant determinants of women’s health care decision making autonomy. The Intra-Class Correlation (ICC) were estimated in the null model to estimate the clustering effect. For model comparison, deviance (-2LLR), Akakie Information Criteria (AIC), and Bayesian Information Criteria (BIC) parameters were used to choose the best-fitted model. Variables with a *p*-value < 0.2 in the bivariable analysis were considered for the multivariable analysis. In the multivariable mixed-effect logistic regression analysis, the Adjusted Odds Ratio (AOR) with 95% Confidence Interval (CI) were reported to declare the strength and significance of the association between women’s decision-making autonomy and independent variables.

**Results:**

In this study, about 81.6% (95% CI: 80.6%, 82.2%) of women have autonomy in making health care decisions. The spatial distribution of women’s autonomy in making health decisions in Ethiopia was non-random (global Moran’s I = 0.0675, *p* < 0.001). The significant hotspot areas of poor women’s autonomy in making health care decisions were found in north Somali, Afar, south Oromia, southwest Somali, Harari, and east Southern Nations Nationalities and Peoples (SNNP) regions. In the mixed-effect logistic regression analysis; being urban (AOR = 1.59, 95% CI: 1.04, 2.45), having secondary education (AOR = 1.60, 95% CI: 1.06, 2.41), having an occupation (AOR = 1.19, 95% CI: 1.01, 1.40) and being from the richest household (AOR = 2.14, 95% CI: 1.45, 3.14) were significantly associated with women autonomy in deciding for health care.

**Conclusions:**

The spatial distribution of women’s autonomy in making the decision for health care was non-random in Ethiopia. Maternal education, residence, household wealth status, region, and maternal occupation were found to influence women’s autonomy. Public health interventions targeting the hotspot areas of poor women autonomy through enhancing maternal occupation and employment is needed to improve women empowerment in making decisions for health care.

## Background

Women autonomy is defined as the ability of women to make choices or decisions within the household relative to their husbands [1, 2]. Women’s autonomy in making decisions for health care is a key public health intervention for better maternal and child health outcomes [3] and is closely linked with the education, residence, and household wealth status of the women [4]. Women’s ability to pursue maternal health care services such as family planning, Antenatal Care (ANC), health facility delivery, Postnatal Care (PNC), child immunization, and seeking health care for their illness depends on women autonomy in making decisions for health care [5–7].

Decision-making autonomy in the use of maternal and child health care plays a major role in women’s health-seeking behavior [8], identified as a key strategy in reducing child and maternal mortality in many resource-poor settings particularly in sub-Saharan Africa [9, 10]. In low- and middle-income countries despite various public health interventions to improve maternal and child health, maternal and child mortality remains a major public health problem [11, 12]. Maternal and child mortality are highly concentrated in low-income countries and is largely due to the low utilization of maternal health services and empowerment of women for their health [13].

Nearly 295,000 women and 5 million children under five died annually worldwide, of which 86% are found in Sub-Saharan Africa (SSA) and Southern Asia [14–17]. The majority of the deaths can be preventable through the utilization of basic maternal and child health care services [15]. Women’s autonomy in making decisions for health care plays a significant role in improving maternal and child health outcomes [18]. Evidences revealed that women’s involvement in making decisions for health care increased the likelihood of maternal and child health care services [13, 19–22].

Despite increasing women’s autonomy in maternal health care as promising strategies to enhance maternal and child health care utilization, women in low-income countries have little control over household assets and participation in health care decisions [22–24]. Women’s autonomy in making decisions are influenced by community norms, culture, gender roles, gender inequality, religion, and other behavioral factors [25, 26]. Previous studies found that maternal age, husband age, maternal education, husband education, household wealth status, parity, residence, and media exposure were significantly associated with women’s autonomy in deciding on health care [27–30].

There are studies conducted on the prevalence and associated factors of women’s decision-making autonomy in Ethiopia [18, 31, 32]. However, in making health care decisions, these findings fail to capture the spatial distribution of women’s autonomy. Therefore, to guide targeted interventions, it has become important to define geographical areas with poor women’s health care decision-making autonomy using Geographic Information Systems (GIS) and Spatial Scan Statistical (SaTScan) analyses. To explore and define the spatial distribution of health care decision-making autonomy of women within a country, GIS and SaTScan-based spatial analysis are crucial. It is necessary to consider areas where women’s autonomy is restricted to having focused interventions in this field to examine the spatial distribution of health care decision-making autonomy. Therefore, this study aimed to investigate the spatial clusters distribution and modelling of health care autonomy among reproductive-age women in Ethiopia.

## Methods

### Data source, population, and sample size

**Fig. 1:**
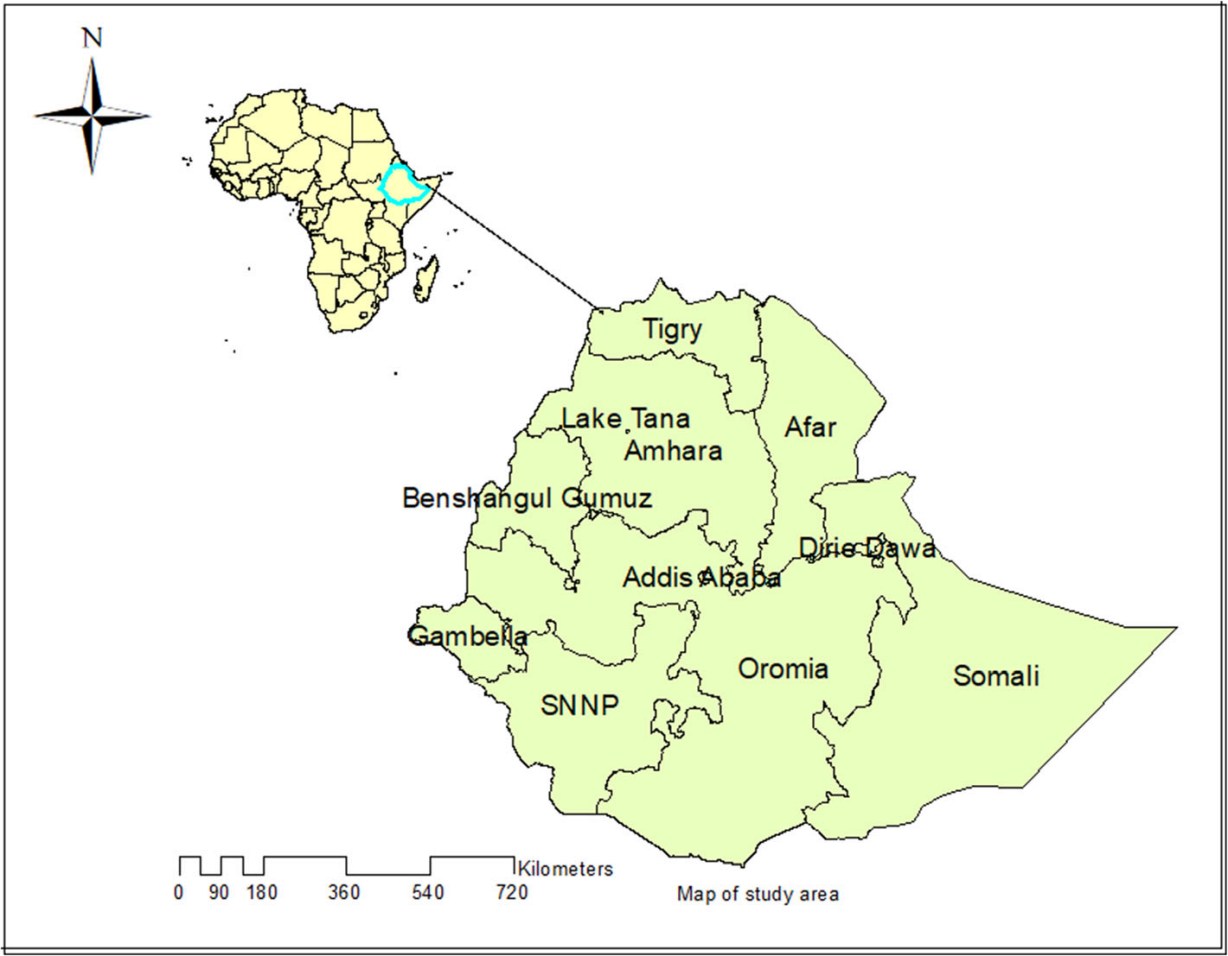
The map of regions of Ethiopia (Figure was generated using ArcGIS version 10.6 statistical software)

This study was based on the most recent Demographic and Health Survey (DHS) of Ethiopia (EDHS 2016). Ethiopia is situated in the horn of Africa and it has nine regions (Afar, Amhara, Benishangul-Gumuz, Gambela, Harari, Oromia, Somali, Southern Nations, Nationalities, and People’s (SNNP) Region and Tigray) and two administrative cities (Addis Ababa and Dire-Dawa) (Fig. 1). A two-stage stratified sampling technique was employed to select the study participants using the 2007 Population and Housing Census (PHC) as a sampling frame. Overall a total of 21 sampling strata have been created. Around 645 Enumeration Areas (EAs) (202 in the urban area) were selected in the first stage and on average 28 households per EA were chosen in the second stage. All currently married reproductive-age women in Ethiopia were the source of population, whereas, all currently married women in the selected EAs were the study population. For this study, the Individual Record data (IR) set was used. A total of 10,223 currently married reproductive age women were included. The detailed sampling procedure and methodology were presented in the full EDHS 2016 report [33].

### Variables of the study

The outcome variable for this study was women’s health decision making autonomy. In EDHS 2016 the question was asked as “person who usually decides on the respondent’s health care?“. The response for this question was respondent alone coded as “1”, jointly with their partner coded as “2”, and partner alone coded as “3”. Then we recode women who take health care decision alone or with their partner were coded 1 while respondents, where their partner alone decides for health care, were coded 0. Where “0” represents a woman with no health care decision making autonomy and “1” represents a woman with health care decision making autonomy. The independent variables considered were maternal age (15–24/25–34/35–49 years), residence (rural/urban), region, maternal occupational status (working/not working), women’s educational status (no/primary/secondary/higher), husband’s educational status (no/primary/secondary/higher), religion (Muslim/orthodox/protestant/other), frequency of watching television (not at all/less than once a week/at least once a week), frequency of listening radio (not at all/less than once a week/at least once a week), frequency of reding newspaper (not at all/less than once a week/at least once a week), household wealth status (poorest/poorer/middle/richer/richest), and covered by health insurance (no/yes).

### Data management and analysis

All the statistics reported in this paper were adjusted for complex survey designs to get a reliable estimate and to draw valid conclusions. STATA version 14, ArcGIS version 10.6, and SaTScan version 9.6 statistical software were used for analysis.

### Spatial analysis

The global spatial autocorrelation using Moran’s index was done to assess whether women’s autonomy in health care decision making was random or non-random. Moran’s index is a spatial statistic that measures the spatial autocorrelation of women’s autonomy in deciding on health care [34]. It produces a single output by taking the entire data sets (proportion of women autonomy in deciding for health care at the cluster level, latitude, longitude, and cluster ID). The global Moran’s I statistical analysis produces the Moran’s I value, Z-score, and *p*-values. Moran’s I value ranges from − 1 to 1 [35]. A value close to 1 shows a strong positive spatial autocorrelation of women’s autonomy in making decisions for health care and a value close to -1 shows a strong negative spatial autocorrelation (opposition between enumeration areas concerning the prevalence of women’s health care decision making autonomy). Moran’s, I value close to 0, indicates the spatial distribution of women autonomy in deciding for health care randomly distributed (independence between EAs). A statistically significant Moran’s I (*p* < 0.05) indicates the spatial distribution for women’s autonomy in deciding for health care is non-random.

To predict the prevalence of health care decision-making autonomy in the un-sampled areas based on sampled EAs, the spatial interpolation method is used. In unobserved areas of Ethiopia, the Kriging spatial interpolation approach was used to predict the prevalence of women’s autonomy of health care decision-making. Kriging is used by forecasting it at unsampled locations (enumeration areas) to generate smooth maps of the outcome (women’s health care decision-making autonomy) and is an optimal interpolation based on regression against observed values of the surrounding data points, weighted according to the spatial covariance values.

The Spatial Scan Statistical (SaTScan) analysis was done to identify significant hot spot areas of women’s autonomy in deciding on health care. The Bernoulli model was employed to identify the statistically significant spatial clusters of health care decision making autonomy of women using Kuldorff’s SaTScan version 9.6 statistical software. Since the elliptical window is inactive in the SaTScan software, we used a circular scanning window that moves across the study area to identify significant hot spot areas. The numbers of cases in each location had Bernoulli distribution and the model required data for cases, controls, and geographic coordinates. Women who are not participating in health care decision making were considered as cases and those who participated in making health care decisions as controls. The default maximum spatial cluster size of < 50% of the population was used as an upper limit, which allowed both small and large clusters to be detected and ignored clusters that contained more than the maximum limit. Selecting the cluster size of 50% of the total population is the default option for the maximum scanning window size and is often used to search the most likely clusters with a higher value of the test significance. Kuldorff’s indicated that a window-sized up to 50% of the population at risk can reduce negative clusters, highly sensitive, avoid missing clusters, and more likely to contain the true significant clusters than the small scanning window.

For each potential cluster, a likelihood ratio test statistic and the *p*-value were used to determine if the number of observed women who are not participating in health care decision making within the potential cluster was significantly higher than expected or not. The scanning window with maximum likelihood was the most likely performing cluster, and the *p*-value was assigned to each cluster using Monte Carlo hypothesis testing by comparing the rank of the maximum likelihood from the real data with the maximum likelihood from the random datasets. The primary and secondary clusters were identified and assigned *p*-values and ranked based on their likelihood ratio test, based on 999 Monte Carlo replications [36].

### Mixed effect logistic regression analysis

The data source for this study was EDHS data. Standard models such as the logistic regression model are not appropriate these models are used for data that has a flat structure but EDHS has hierarchical nature (data collected at individual and community level). This implies that there is a need to take into account the between cluster variability by using advanced models such as mixed-effect binary logistic regression analysis. Therefore, a mixed effect logistic regression model (both fixed and random effect) was fitted. By fitting the standard logistic regression and mixed-effects logistic regression models, deviance (-2LLR), Akakie Information Criteria (AIC), and Bayesian Information Criteria (BIC) were used as a model comparison parameter. The Intra-class Correlation Coefficient (ICC), Likelihood Ratio (LR) test, and Median Odds Ratio (MOR) were done to assess the clustering effect and for assessing model fitness. Variables with a *p*-value < 0.2 in the bi-variable analysis were considered in the multivariable mixed-effect logistic regression model. The Adjusted Odds Ratios (AOR) with a 95% Confidence Interval (CI) and *p*-value ≤ 0.05 in the multivariable model were used to declare statistically significant factors associated with women’s autonomy in making health care decisions.

### Ethics consideration

Permission to get access to the data was obtained from the measure DHS program online request from http://www.dhsprogram.com.website and the data used were publicly available with no personal identifier.

## Results

### Background characteristics of reproductive‐age women

A total of 10,223 currently married women were included in the study. Of these, 81.6%% (95% CI: 80.6, 82.2%) of married women took decisions on their health care alone or jointly with their husband. About 6815 (79.6%) of rural women and 1508 (91%) of urban women were autonomous in making decisions for their health care. Regarding region, more than three-fourth (92.8%) of women in Addis Ababa and 91.6% of women in Harari took decisions on their health care alone or jointly with their husbands. Women’s autonomy in making decisions for their health care increased by education status, from 79.2% among women who did not attain formal education to 92.2% among women who attained a higher level of education. The proportion of women who participated in making decisions for their health care among women whose husbands attained a higher level of education, those in the richest household wealth status, those who have media exposure (radio, newspaper, and television) were higher compared to their counterparts (Table 1).

**Table 1:** Percentage distribution of women autonomy in making decisions for health care currently married women by women characteristics in Ethiopia, 2016

Variables	Women autonomy in making decision for health care	
	**No (%)**	**Yes (%)**
**Residence**		
Rural	1749 (20.4)	6815 (79.6)
Urban	150 (9)	1256 (91)
**Region**		
Tigray	104 (15.8)	553 (84.2)
Afar	27 (28.8)	68 (71.2)
Amhara	313 (13.0)	2100 (87.0)
Oromia	805 (20.2)	3182 (79.8)
Somali	78 (24.1)	245 (75.9)
Benishangul	23 (20.3)	90 (79.7)
SNNPRs	507 (23.3)	1666 (76.7)
Gambella	6 (20.6)	23 (79.6)
Harari	2 (8.4)	22 (91.6)
Addis Ababa	25 (7.2)	329 (92.8)
Dire-Dawa	7 (15.6)	43 (84.4)
**Education status**		
No education	1302 (20.8)	4951 (79.2)
Primary	509 (17.6)	2386 (82.4)
Secondary	55 (8.5)	599 (91.5)
Higher	33 (7.8)	599 (92.2)
**Religion**		
Orthodox	608 (14.7)	3531 (85.3)
Muslim	762 (21.5)	2777 (78.5)
Catholic	9 (12.5)	66 (87.5)
Protestant	458 (20)	1831 (80)
Others	61 (36.1)	108 (63.9)
**Maternal age (years)**		
15–24	458 (19.9)	1839 (80.1)
25–34	746 (16.8)	3705 (83.2)
≥ 35	695 (20)	2779 (80)
**Husband education**		
No	953 (20.3)	3732 (79.7)
Primary	694 (18.4)	3078 (81.6)
Secondary	150 (15.4)	825 (84.6)
Higher	89 (12.6)	623 (87.4)
**Wealth status**		
Poorest	463 (23.7)	1489 (76.3)
Poorer	467 (22.5)	1607 (77.5)
Middle	391 (19.0)	1666 (81.0)
Richer	365 (18.3)	1634 (81.7)
Richest	213 (9.9)	1927 (90.1)
**Frequency of reading newspaper**		
Not at all	1827 (19.5)	7531 (80.5)
Less than once a week	55 (8.7)	585 (91.3)
At least once a week	17 (7.6)	208 (92.4)
**Frequency of listening to the radio**		
Not at all	1410 (19.8)	5711 (80.2)
Less than once a week	285 (18.2)	1278 (81.8)
At least once a week	204 (13.3)	1334 (86.7)
**Frequency of watching television**		
Not at all	1550 (19.7)	6328 (80.3)
Less than once a week	225 (20.3)	885 (79.7)
At least once a week	124 (10)	1111 (90)
**Covered by health insurance**		
No	1821 (18.8)	7891 (81.2)
Yes	78 (15.3)	433 (84.7)
**Respondent working status**		
Not working	1458 (20.7)	5602 (79.3)
Working	441 (13.9)	2722 (86.1)
**Overall prevalence**	18.6% (95% CI: 17.8, 19.3)	81.4% (95% CI: 80.6, 82.2)

### Spatial distribution of health care autonomy among reproductive‐age women

**Fig. 2:**
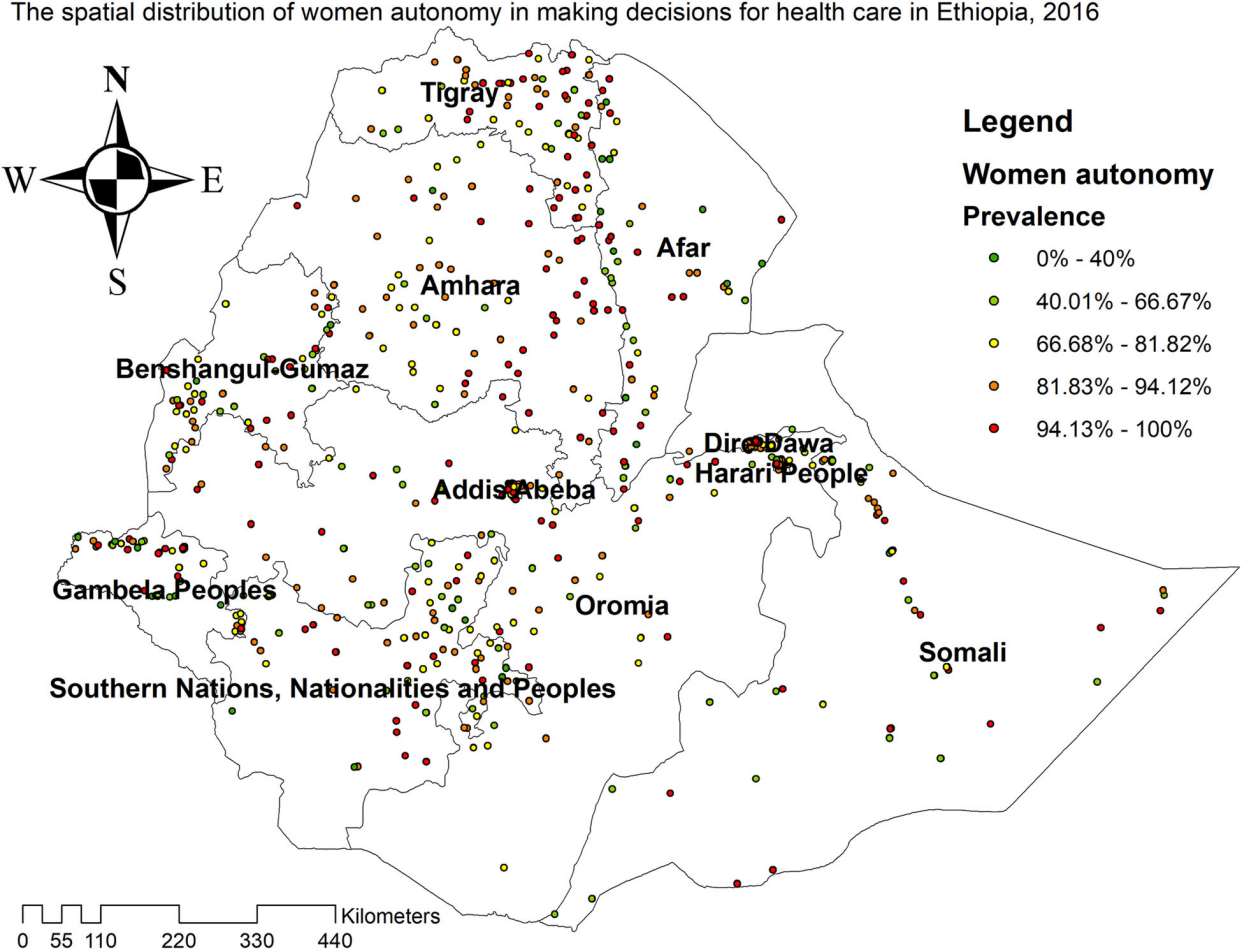
The spatial distribution of women autonomy in making decisions for health care among reproductive age women in Ethiopia, 2016 (Figure was generated using ArcGIS version 10.6 statistical software)

**Fig. 3:**
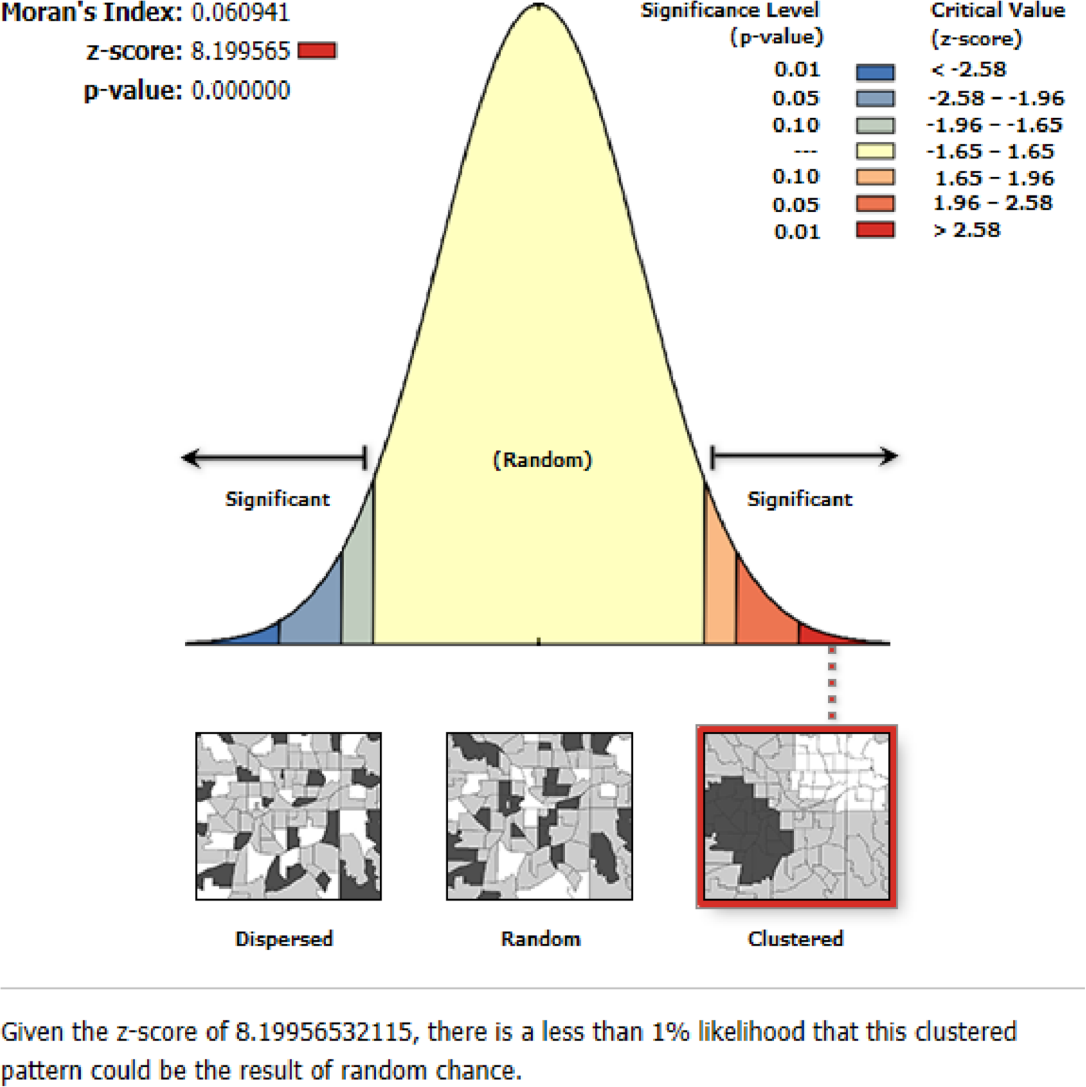
Global spatial autocorrelation analysis of women autonomy in making health care decision in Ethiopia, 2016 (was generated using ArcGIS version 10.6 statistical software)

The red color on the map indicates areas with a high percentage of women who were autonomous in making decisions for their health care. Whereas, the green one indicates a low percentage of women who were autonomous in making decisions for their health care. The high prevalence of women who were autonomous in making decisions for their health care was found in Addis Ababa, Tigray, and Amhara regions whereas, a low percentage of women’s health care autonomy was found in the Somali, West Benishangul, and East SNNP regions (Fig. 2). The global spatial autocorrelation analysis showed that the spatial distribution of health care making autonomy of women was non-random in Ethiopia (global Moran’s I = 0.0675, *p* *<* 0.001) (Fig. 3).

### Kriging interpolation analysis

**Fig. 4:**
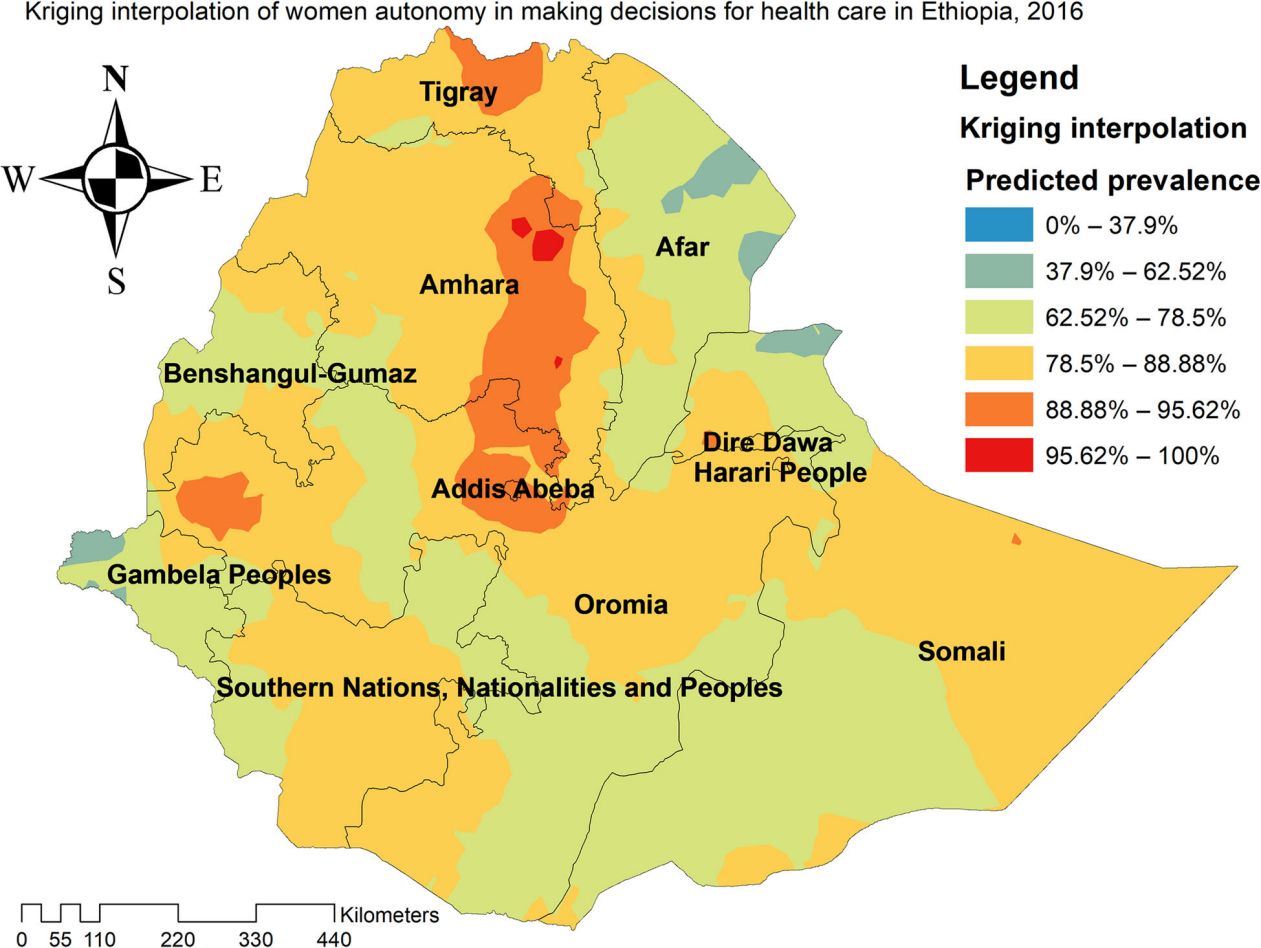
The spatial interpolation of women autonomy in making health care decisions in Ethiopia, 2016 (Figure was generated using ArcGIS version 10.6 statistical software)

The predicted highest prevalence of women who were autonomous in making decisions for health care was detected in the central Amhara, north Oromia, and east Somali regions. In contrast, areas with the lowest prevalence of women autonomy in making decisions for making health care were detected in east Afar, northeast Somali, west SNNPRs, and west Gambella regions (Fig. 4).

### Spatial scan statistical analysis

**Fig. 5:**
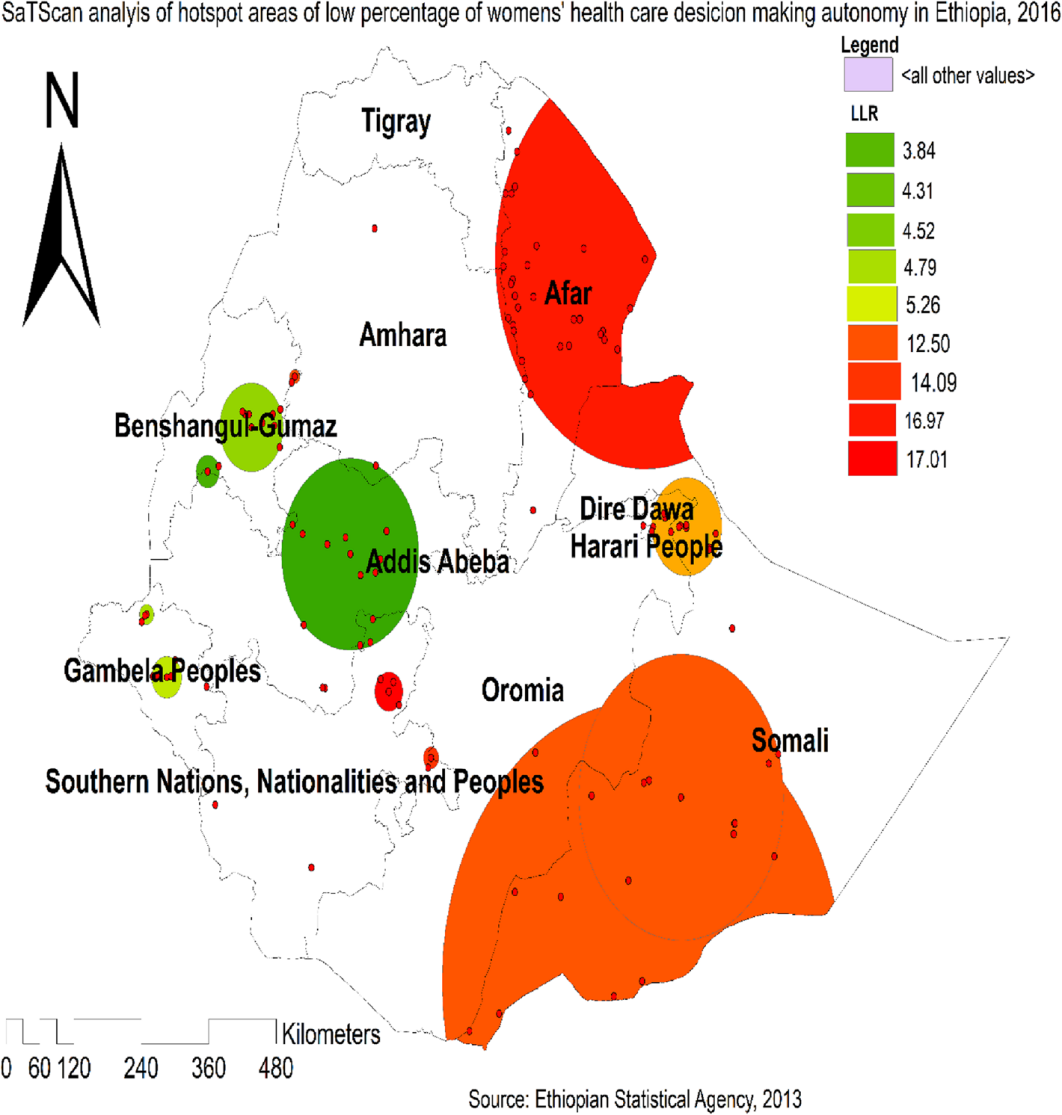
The Spatial Scan Statistical analysis of women autonomy in making decisions for health care in Ethiopia, 2016 (Figure was generated using ArcGIS version 10.6 and SaTScan version 9.6 statistical software)

A total of 165 significant clusters were identified within 3 spatial windows. Of these, 78 clusters were primary (most likely clusters) located in the north Somali and entire Afar regions of Ethiopia. The cluster’s spatial window was centered at 9.956410 N, 38.964524 E with a radius of 122.65 km, a Relative Risk (RR) of 1.15, and Log-Likelihood Ratio (LRR) of 17.1, at *p* < 0.01. This means married women within the spatial window had 1.15 times less likely to be participated in making health care decisions compared to women outside the spatial window (Table 2). The secondary clusters were identified in the south Oromia, southwest Somali, Harari, and east SNNPRs regions (Fig. 5).

**Table 2:** The spatial scan statistical analysis of women autonomy in making decisions for health care among women in Ethiopia, 2016

Cluster	Enumeration area(cluster)identified	Coordinate/radius	Population	Case	RR	LLR	*p*-value
1 (78)	572, 423, 510, 267, 310, 10, 201, 637, 460, 624, 350, 121, 229, 482, 287, 112, 464, 303, 90, 144, 463, 295, 274, 532, 153, 170, 402, 247, 91, 369, 560, 509, 15, 428, 582, 11, 626, 639, 414, 339, 305, 108, 107, 155, 635, 31, 100, 211, 19, 40, 330, 293, 159, 195, 59, 314,264, 302, 487, 176, 645, 110, 61, 225, 608, 451, 539, 206, 145, 617,475, 261, 252, 484, 236, 83, 147, 353	(9.956410 N, 38.964524 E)/122.65 km	669	619	1.15	17.1	0.0001
2(70)	423, 572, 510, 112, 463, 464, 274, 144, 287, 532, 153, 170, 91, 90, 247, 11, 626, 339, 15, 582, 107, 108, 305, 414, 509, 402, 560, 639, 31, 428, 267, 635, 100, 303, 155, 19, 195, 211, 159, 314, 293, 59, 330, 487, 645, 302, 264, 608, 110, 145, 225, 61, 451, 539, 350, 121, 40, 475, 261, 201, 10, 252, 147, 236, 229, 83, 353, 310, 482, 517	(9.739794 N, 38.793592 E)/103.33 km	593	549	1.15	16.97	0.001
3 (17)	616, 354, 617, 18, 410, 345, 611, 460, 496, 545, 254, 591, 176, 478,10, 267, 368	(9.555410 N, 40.326165 E)/34.04 km	205	198	1.19	14.09	0.05

### Determinants of health care decision making autonomy

#### Model comparison

AIC, BIC, and deviance were used for model comparison, and the mixed effect logistic regression model was the best fitted for the data since it had the lowest deviance value (Table 3). The ICC value in the null model was 0.26 (95% CI: 0.21, 0.31), MOR was 2.76 and the Log-likelihood Ratio (LR) test was (X^2^ (01) = 293.29, *p* < 0.001), indicates that the mixed effect logistic regression model was the best fit over the basic model.

**Table 3:** Model comparison parameters for comparing standard logistic regression and mixed effect logistic regression model

Model comparison	AIC	BIC	Deviance
The standard logistic regression model	5638.43	5853.6	5574.43
The mixed-effect logistic regression model	5468.07	5689.92	5402.06

*AIC* Akaike Information Criteria, *BIC* Bayesian Information Criteria

In the multivariable mixed-effect logistic regression analysis; place of residence, region, woman’s educational status, woman’s occupation, and household wealth index were significantly associated with women’s autonomy for health care decision making. Urban women were 1.59 times (AOR = 1.59, 95% CI: 1.04, 2.45) higher odds of participating in making decisions for their health care compared to rural women. Women residing in Tigray, Amhara, Oromia, and Harari region were 2.41 (AOR = 2.41, 95% CI; 1.43, 4.04), 3.02 (AOR = 3.02, 95% CI; 1.83, 4.99), 1.72 (AOR = 1.72, 95% CI: 1.07, 2.78) and 4.17 (AOR = 4.17, 95% CI: 2.14, 8.14) times higher odds of participating in making their own health care decisions compared to those women residing in Afar region, respectively. The odds of participating in making decisions for their health care among women who attained secondary education were 1.6 times (AOR = 1.60 (95% CI: 1.06, 2.41) higher than women with no formal education. The odds of participating in making their own health care decisions among women who were working were 1.19 times (AOR = 1.19 (95% CI: 1.01, 1.40)) higher compared to women who had no occupation/not working. Women from the richest household had 2.14 times (AOR = 2.14 (95% CI: 1.45, 3.14)) higher odds of participating in making their own health care decisions compared to women from the poorest household (Table 4).

**Table 4:** The bivariable and multivariable mixed effect logistic regression analysis of determinants of women autonomy in making decisions for health care in Ethiopia, 2016

Variables	Crude Odds Ratio (COR) with 95% CI	Adjusted Odds Ratio (AOR) with 95% CI
**Residence**		
Rural	1	1
Urban	2.87 [2.17, 3.79]	1.59 [1.04, 2.45] ^**^
**Region**		
Afar	1	1
Tigray	3.00 [1.79, 5.01]	2.41 [1.43, 4.04] ^**^
Amhara	3.62 [2.21, 5.92]	3.02 [1.83, 4.99] ^*^
Oromia	1.95 [1.22, 3.12]	1.72 [1.07, 2.78] ^*^
Somali	1.38 [0.84, 2.27]	1.32 [0.82, 2.14]
Benishangul	1.76 [1.05, 2.95]	1.50 [0.90, 2.52]
SNNPRs	1.64 [1.02, 2.62]	1.44 [0.89, 2.33]
Gambella	1.40 [0.81, 2.42]	1.09 [0.63, 1.89]
Harari	6.09 [3.15, 11.77]	4.17 [2.14, 8.14] ^*^
Addis ababa	5.81 [3.19, 10.57]	1.85 [0.95, 3.62]
Dire Dawa	2.54 [1.44, 4.48]	1.39 [0.78, 2.50]
**Maternal age (years)**		
15–24	1	1
25–34	1.21 [1.01, 1.45]	1.21 [0.99, 1.46]
≥ 35	1.06 [0.88, 1.29]	1.06 [0.86, 1.31]
**Woman’s education status**		
No education	1	1
Primary	1.19 [1.00, 1.41]	1.12 [0.92, 1.36]
Secondary	2.09 [1.48, 2.96]	1.60 [1.06, 2.41] ^*^
Higher	2.74 [1.70, 4.41]	1.55 [0.88, 2.73]
**Respondent working status**		
Not working	1	1
Working	1.38 [1.18, 1.61]	1.19 [1.01, 1.40] ^*^
**Frequency of reading newspaper/ magazine**		
Not at all	1	1
Less than once a week	1.82 [1.28, 2.57]	1.19 [0.81, 1.75]
At least once a week	2.67 [1.34, 5.33]	1.76 [0.85, 3.64]
**Frequency of listening radio**		
Not at all	1	1
Less than once a week	1.18 [0.95, 1.47]	1.02 [0.80, 1.29]
At least once a week	1.25 [1.00, 1.56]	0.98 [0.76, 1.25]
**Frequency of watching television**		
Not at all	1	1
Less than once a week	0.95 [0.74, 1.21]	0.65 [0.49, 0.86]
At least once a week	1.99 [1.52, 2.61]	0.78 [0.54, 1.12]
**Household wealth status**		
Poorest	1	1
Poorer	1.21 [0.96, 1.51]	1.04 [0.82, 1.32]
Middle	1.49 [1.17, 1.90]	1.28 [0.99, 1.66]
Richer	1.40 [1.09, 1.79]	1.18 [0.91, 1.55]
Richest	3.19 [2.46, 4.13]	2.14 [1.45, 3.14] ^*^
**Covered by health insurance**		
No	1	1
Yes	1.46 [0.99, 2.14]	1.08 [0.73, 3.14]
**Husband education status**		
No education	1	1
Primary	1.09 [0.92, 1.30]	0.97 [0.81, 1.16]
Secondary	1.16 [0.89, 1.50]	0.74 [0.55, 1.00]
Higher	1.64 [1.21, 2.23]	0.85 [0.59, 1.21]

*p-value < 0.05, **p-value < 0.01, *CI* Confidence Interval, *COR* Crude Odds Ratio, *AOR* Adjusted Odds Ratio

## Discussion

In this study, the prevalence of women who were autonomous in making health care decisions was 81.6% (95% CI: 80.6, 82.2). This is higher than studies reported in Dabat [37], Northern Ethiopia [38], Southern Ethiopia [18], and Ghana [27]. This may be due to the discrepancy in the research’s scope, as the current study was focused on data at the national level that was represented at the level of region and residence. Besides, the disparity may be due to the difference in the research period, since the current study was based on data from EDHS 2016 whereas the study in Ghana was based on data from GDHS 2014. Therefore, because of the introduction of many policies and services aimed at increasing women’s empowerment, women’s health care decision-making autonomy has been increased over time [39, 40].

The spatial analysis showed that in Ethiopia, the spatial distribution of women autonomy in decision-making was non-random (Moran’s I = 0.0675, *p* < 0.001). In north Somali, entire Afar, southern Oromia, southwest Somali, Harari, and eastern SNNP regions, the hotspot areas of poor women’s autonomy in decision-making for health care were established. The high rate of maternal and child mortality in these areas has confirmed this finding, as maternal and child mortality is closely related to the low empowerment of women in society [41]. The potential explanation may be because more rural residents are in the border areas of Afar, Somali, SNNPRs, and the Oromia regions and thus have poor access to education, media exposure, and health care facilities. This may also lead to a decreased degree of women’s autonomy in decision-making on health care [42, 43]. Besides, this may be due to the disparity in the socio-economic, behavioral, cultural, gender position, and status of women in different areas of the country across communities [44].

In the multivariable mixed-effect logistic regression analysis; place of residence, region, woman’s educational status, woman’s occupation, and household wealth index were significantly associated with women’s autonomy for health care decision making. Urban women had higher odds of health care decision making autonomy compared to rural women. This is consistent with studies in developing countries [22], and South Asia [45]. The potential explanation may be that urban women have greater access to the media and aware of their right to free choice as opposed to rural women, this could help women decide on their health care individually or jointly with their husband [46]. Compared to women residing in the Afar region, women living in the Amhara, Oromia, Tigray, and Harari regions had higher odds of autonomy in making health care decisions. This may be due to the relatively good household wealth of the Amhara, Tigray, Harari, and Oromia regions, and more urbanized. They are also more likely to access qualified health practitioners, health services, health information, and education, which may improve women’s empowerment in decision-making [47, 48].

Women who attained secondary education had higher odds of health care decision making autonomy compared to women with no formal education. This is in line with study findings in Ethiopia [49], Nepal [6], and Sub-Saharan Africa [50]. This could be the fact that educated women are more likely to take part in decision making in their health care because they exercised gender equality [51]. Besides, education enhances self-confidence, their awareness towards health care decision and build up women’s capacity in developing their own decisions [6]. The association between women’s occupation and autonomy in making decisions for health care was positively associated. This finding is consistent with a study done in South Asia [45], in which working women are more likely to participate in health care decision making than women who are not working. This is because women who are working had higher income, and contact with different individuals, this could increase women’s economic independence, access to information and challenges the beliefs of men’s dominance in making decisions [51].

Household wealth status was found a significant predictor of health care decision making autonomy of women in this study. This study found that women from the richest household wealth had a higher chance of participating in health care decision making than women from the poorest household. This is in line with studies in southern Ethiopia [18], and Developing countries [22]. The possible justification could be due to women in the richer household are more of employed, increased level of self-confidence, and access to information, this could improve women’s involvement in making health care decisions [22].

## Conclusions

The spatial distribution of women’s autonomy in making decisions for health care was non-random in Ethiopia. Residence, maternal education, household wealth status, region, and maternal occupation were significant factors affecting the ability of women to take part in making decisions for their health care. Therefore, attention should be given to those women who do not attend, hotspot areas of poor women autonomy and rural residents through public health programs target women education and employment. The finding of this study should be interpreted in light of limitations. First, because of the cross-sectional nature of the Ethiopian demographic and health survey, which makes it impossible to draw the temporal/causal relationship between the variables, only the association between variables can be drawn. Secondly, the data were obtained from the respondent’s self-report and this has the tendency of recall and social desirability bias, therefore, these may underestimate or overestimate the true association between women health care decision making autonomy and the independent variables. Thirdly, since this study is secondary data analysis, we did not include variables such as community literacy, community beliefs, and attitude towards women empowerment that might relate to women’s health care decision making autonomy. Overall, these issues could affect the internal and external validity of these findings. Therefore, to overcome the limitations of this study further in-depth research into community attitude, beliefs, practice and cultural perception towards women health care decision autonomy is needed autonomy is needed for a better understanding of women health care autonomy.

## Data Availability

Data is available online and you can access it from www.measuredhs.com.

## Abbreviations

AIC: Akaike Information Criteria
AOR: Adjusted Odds Ratio
BIC: Bayesian Information Criteria
CI: Confidence Interval
CSA: Central Statistical Agency
DHS: Demographic Health Survey
EA: Enumeration Area
EDHS: Ethiopian Demographic Health Survey
GIS: Geographic Information System
GDHS: Ghana Demographic and Health Survey
ICC: Intra-class Correlation Coefficient
LR: Likelihood Ratio
LLR: Log-Likelihood Ratio
MOR: Median Odds Ratio
PHC: Population and Housing census
SNNPRs: Southern Nations and Nationality People Regional state
